# Simulated chainsaw injury bone fracture data

**DOI:** 10.1016/j.dib.2022.107872

**Published:** 2022-01-24

**Authors:** Geoffery T. Desmoulin, Theodore E. Milner

**Affiliations:** GTD Scientific Inc., 2037 MacKay Avenue, North Vancouver V7P 2M8, Canada

**Keywords:** Chainsaw, Forearm, Laceration, Bone fracture

## Abstract

Forensic analysis is often required to determine the cause of an injury. Data for this purpose were acquired by simulating an injury to a limb inflicted by a chainsaw. A surrogate forearm was constructed from gel and a bone simulant. A series of 10 arms were severed under different conditions of chainsaw operation, arm position and arm resistance. The bone fracture force was determined from force records acquired with a force plate which supported the test rig holding the arm. A break wire in the arm signalled the time of fracture. The data set constitutes the reaction force registered by the force plate and the break wire signal. Both signals were digitally sampled at 1000 Hz. Photographs of the proximal portion of each severed arm were taken with a digital camera and are included in the data set. This data set is of interest to forensic investigators considering injuries inflicted by power tools. The data provide a benchmark for planning tests of simulated injuries. They can also be compared to experiments carried out on cadaveric specimens to determine the accuracy of such simulations. This article is being submitted as a co-submission with the following article, G.T. Desmoulin, T.E. Milner, Methodology for determining accidental versus intentional injury afflicted by a chainsaw. Forensic Science International. https://doi.org/10.1016/j.forsciint.2021.110993.

## Specifications Table

**Table:** 

Subject	Materials Mechanics
Specific subject area	Bone fracture patterns inflicted by chainsaw
Type of data	Force time series, digital images
How data were acquired	Digital sampling of force plate signal with National Instrument DAQ and digital photography
Data format	Raw
Parameters for data collection	Forceful swing of chainsaw Free fall drop of chainsaw
Description of data collection	Force plate signals were acquired with a National Instruments data acquisition system sampling at 1000 Hz. Photographs were taken with a Canon 7D camera.
Data source location	Institution: GTD Scientific, Inc. City/Town/Region: North Vancouver, British Columbia Country: Canada
Data accessibility	Repository name: Mendeley Data Data identification number: DOI:10.17632/ctm2pbjpv4.1 Direct URL to data: https://data.mendeley.com/datasets/ctm2pbjpv4/1
Related research article	G.T. Desmoulin, T.E. Milner, Methodology for determining accidental versus intentional injury afflicted by a chainsaw, Forensic Science International 328 (2021) 1-4. https://doi.org/10.1016/j.forsciint.2021.110993.

## Value of the Data

• These data show the type of bone fracture pattern expected from a chainsaw injury which is useful in forensic investigations of power tool injuries.
• These data are of value to forensic investigators and clients who hire forensic investigators for cases involving injuries inflicted by power tools.
• These data provide validation of the capacity for high-density foam to represent the mechanical properties of human bone and, thereby, give forensic investigators an additional tool to use for forensic tests.

## 1 Data Description

ChainsawData_25May2018.xlsx is an Excel file containing 10 worksheets. Each worksheet contains a data set from one chainsaw laceration test. There are three columns. The first column is the time relative to contact of the chainsaw with the break wire embedded in the surrogate arm. The second column is the break wire signal measured in volts. The third column is the reaction force of the test rig plus the force applied by the chainsaw measured in newtons (N). The raw signals in volts from loads cells located at the four corners of the force plate were multiplied by their respective calibration factors and summed to get the reaction force. Each worksheet name and a short description of the corresponding test is listed below. Photographs were taken of the lacerated surrogate arm showing the bone fracture pattern for each test. The corresponding file names for the photographs are listed with each worksheet name (Table 1).

**Table 1:** Worksheet name and description for photographs of injury patterns.

Worksheet Name	Description	Photos
20180514T135708	chainsaw forcefully strikes arm supported on both sides of cut, arm in supinated posture	IMG_0174.JPG, IMG_0175.JPG, IMG_0176.JPG
20180514T140557	chainsaw forcefully strikes arm supported on both sides of cut, arm in supinated posture	IMG_0322.JPG, IMG_0323.JPG
20180514T142652	chainsaw forcefully strikes arm supported on both sides of cut, arm in pronated posture	IMG_0184.JPG, IMG_0186.JPG
20180514T141957	chainsaw forcefully strikes arm supported on both sides of cut, arm in pronated posture	IMG_0327.JPG, IMG_0328.JPG, IMG_0329.JPG
20180514T144421	chainsaw drops on arm supported on both sides of cut, arm in supinated posture	IMG_0190.JPG, IMG_0191.JPG
20180514T143350	chainsaw drops on arm supported on both sides of cut, arm in supinated posture	IMG_0333.JPG, IMG_0334.JPG, IMG_0335.JPG, IMG_0336.JPG
20180514T151350	chainsaw drops on arm supported on both sides of cut, arm in pronated posture	IMG_0197.JPG, IMG_0198.JPG, IMG_0199.JPG
20180514T144544	chainsaw drops on arm supported on both sides of cut, arm in pronated posture	IMG_0340.JPG, IMG_0341.JPG, IMG_0342.JPG, IMG_0343.JPG
20180514T150823	chainsaw drops with trigger released on arm supported on both sides of cut, arm in supinated posture	IMG_0347.JPG, IMG_0348.JPG, IMG_01349.JPG, IMG_0350.JPG
20180514T152754	chainsaw drops on arm supported on one side of cut, arm in supinated posture	IMG_0358.JPG, IMG_0359.JPG, IMG_0360.JPG, IMG_0361.JPG, IMG_0363.JPG, IMG_0363.JPG

## 2 Experimental Design, Materials and Methods

A surrogate arm was constructed using 250-bloom gelatin (Holly North Production Supplies, Burnaby, BC, Canada) with bones cast from high-density foam “bone simulant” (Coast Fiber-Tek Products Ltd., Burnaby, BC, Canada) in a mold, which matched anthropometric measurements for the ulna and radius of a human male, 180 cm in height. In all but one of the experiments, the arm was supported on either end by a wooden test rig, mounted on a force plate. The experiment consisted of lacerating the arm by cutting through it with a chainsaw (Model 445, 18-inch blade, Husqvarna Group, Stockholm and Husqvarna, Sweden). Two modes of cutting were compared. The first mode, which was referred to as ‘intentional’, had the chainsaw operator deliberately push down on the chainsaw to sever the arm midway between the two supports. The second mode, referred to as ‘unintentional’, had the chainsaw operator allow the chainsaw to drop onto the arm without adding force to the weight of the chainsaw, i.e. without pushing down. In one additional test, the chainsaw operator released the trigger on the chainsaw before letting it drop so that the blade was not powered, but still rotating due to its momentum, as it contacted the arm. In a final test, the arm was supported on one side only, so that the arm did not offer any resistive force to the chainsaw. Digital photographs were taken with a Canon 7D camera (5184 × 3456 pixel resolution) of the proximal portion of the severed arm for visual analysis of the bone fracture pattern [1].

In all tests, the force plate measured the reaction force. The reaction force consisted of the weight of the test rig and the force applied by the chainsaw. A break wire embedded in the arm was used to signal the time at which the chain saw severed the arm. The reaction force and the break wire signal were sampled at 1000 Hz with a National Instruments DAQ. The data files contain the reaction force, as measured in N, beginning 1.001 s prior to the break wire being severed until 4.999 s after the break wire was severed, along with the break wire signal in V [1].

## Ethics Statement

No ethics approval was required since the experiments did not involve testing with human or animal subjects. All testing was carried out on purely mechanical rigs. The acquired data have not been altered in any way other than being calibrated in appropriate units for analysis.

## CRediT Author Statement

**Geoffery Desmoulin:** Conceptualization, Methodology, Data acquisition; **Theodore Milner**: Data curation, Writing.

## Declaration of Competing Interest

(1) No third-party financial support was provided for the work this article;

(2) Both authors are employees of GTD Scientific, Inc.;

(3) Theodore Milner has been paid by GTD Scientific, Inc. for preparing this article;

The authors declare that they have no known competing financial interests or personal relationships which have or could be perceived to have influenced the work reported in this article.
